# Development and Implementation of a Nutritional Education Program Aimed at Improving the Integration Process of Young Orphan Refugees Newly Arrived in Portugal

**DOI:** 10.3390/nu15020408

**Published:** 2023-01-13

**Authors:** Bárbara Oliveira, Manuel Bicho, Ana Valente

**Affiliations:** 1 ATLANTICA—University Institute, 2730-036 Barcarena, Portugal; 2 Ecogenetics and Human Health Research Group, Environmental Health Institute (ISAMB), Faculty of Medicine (FMUL), University of Lisbon, 1649-028 Lisbon, Portugal; 3 Scientific Research Institute Bento da Rocha Cabral (IICBRC), 1250-047 Lisbon, Portugal

**Keywords:** refugee, orphan, adolescent, nutritional status, food literacy

## Abstract

(1) Background: Refugees are a population group at imminent risk of death, being forced to migrate to countries with different cultures. Many of the refugees are at great risk of malnutrition, especially adolescent orphans. The aim of the study was to establish a nutritional and food education program to improve the integration process of young orphan refugees newly arrived in Portugal. (2) Methods: A nutrition and food education program with nine sessions of food and nutrition education over 12 weeks was carried out by a nutritionist from March to June 2016, in 15 young residents of the Reception Center for Refugee Children. The program included the application of a nutritional knowledge questionnaire, an anthropometric assessment, and the collection of data on food habits and lifestyle. The evaluation of the program was carried out by comparing the initial and final scores of the nutritional knowledge questionnaire. (3) Results: There was an improvement in nutritional knowledge among the adolescents, and a direct relationship between attendance at the sessions and improvement of this knowledge was found. Non-significant changes were observed in some anthropometric measurements between the beginning and the ending of the program. (4) Conclusions: This food education program contributed to a better understanding by young orphan refugees newly arrived in Portugal of the foods available in Portugal and of the Portuguese gastronomy.

## 1. Introduction

Adolescence is the period of an individual’s life, according to the World Health Organization (WHO), between the ages of 10 and 19 [1] that presents itself as a phase of rapid body and psychosocial growth. It is a period of transition between childhood and adulthood, characterized by body changes and the development of the individual’s mental, emotional, and social dimensions [2]. In addition to physical and sexual maturation, adolescence is an important stage in psychological development, as it begins to lead the young person towards social and economic independence, and it is at this point that the development of the individual’s identity and the acquisition of personal skills take place [3,4]. It is a time of enormous growth potential and of considerable risk, during which social contexts exert a powerful influence on the person’s experience. The family, school, health services and social institutions have a responsibility to promote healthy growth and therefore have a strong impact on the development of the individual and, consequently, on society [1]. The acquisition of healthy eating habits is established during childhood and adolescence and may be maintained throughout life [5,6]. It is therefore important that at this stage of life, young people gain the ability to voluntarily make healthy food choices that lead to a state of good nutrition and consequent well-being. Any and every educational strategy to achieve this and to guarantee the acquisition of adequate nutritional knowledge is a Nutritional Education activity. Bad eating habits and lack of knowledge are the main reasons for malnutrition problems among adolescents. As adolescents are the future of any country, their nutritional status is critical to the well-being of society. The WHO therefore advocates the implementation of awareness programs among the younger population and emphasizes its importance in the health of the population in general [7]. Today, our world is faced with wars and, consequently, forced displacements, which end up destroying the social structure that is considered normal for an individual, forcing him to introduce himself into new cultures and communities different from those in which he grew up and lived throughout his life [8]. In this way, complex support work is required for integration at all levels into the new society to which individuals are displaced to. Populations that are forced to move from one country to another and, especially, those who move from less developed to more developed countries, go through a nutritional transition process that is associated with changes in their health due to changing social environments, food, physical activity, and access to health care. Scientific evidence [9,10] points to the fact that the eating habits of the displaced populations are significantly more inadequate than the eating habits of the general population of the host country, so there is a greater risk of a decrease in the state of health of these populations due to an inadequate diet and all the other difficulties they face in the new country [11]. The main difficulties that displaced populations experience are lack of access to adequate health care, language difficulties, financial difficulties resulting from unemployment or low incomes, and cultural differences. These barriers will end up in the lack of access to safe and healthy food, resulting in malnutrition and difficulty in maintaining an adequate health status [12]. Refugees are a particularly vulnerable category of these populations, as they move from their homeland to temporary shelter camps or Reception Centers in developed countries that offer a more industrialized Western diet than they were used to at home [13]. A refugee is any person who, because of justified fears of being persecuted on account of his race, religion, nationality, membership of a particular social group or political opinion, is outside his country of origin and who, because of said fears, cannot or does not want to return to it [14]. Many of the refugees who arrive in Portugal are under the age of 18; therefore, the process of achieving autonomy will have to be carried out in this new context, making this stage of the young individual’s life difficult. As part of the work carried out by the Office of the United Nations Food and Agriculture Organization (FAO) in Portugal with the Community of Portuguese-Speaking Countries (CPLP) and in order to promote the eradication of hunger and malnutrition and guarantee food security and nutrition for all, a Food and Nutrition Education project was developed with young refugees who were under the shelter of the Casa de Acolhimento para Crianças Refugiadas (CACR) of the Portuguese Council for Refugees (CPR). The aim of the study was to establish a nutritional and food education program to improve the integration process of young orphan refugees newly arrived in Portugal.

## 2. Materials and Methods

### 2.1. Study Design

A nutrition and food education program with 9 sessions of food and nutrition education over 12 weeks was carried out by a nutritionist from March to June 2016 at the CACR under the protocol signed between FAO and CPR with activities previously defined by Atlântica, the FAO Office in Portugal, and with the CPLP. The study was part of the work carried out by the FAO Office in Portugal and with the CPLP, to promote, in terms of partnerships and dissemination of information, its mandate to eradicate hunger and malnutrition and guarantee food and nutritional security for all. Thus, an intervention plan for food education was developed by a nutritionist for young refugees who were at the Reception CACR of the CPR. This plan aimed to ensure that this vulnerable group of the population displaced to Portugal was educated on health and food safety, in addition to promoting their prosperous inclusion in Portuguese culture and society. The authors obtained authorization within the scope of the collaboration project between Atlântica, the FAO Office in Portugal, and the CACR. An activity plan was previously prepared between the institutions to outline all the activities to be carried out during the study period.

### 2.2. Study Population

The population group studied was composed of the young orphan refugees residing in the CACR of the CPR. Thus, the entire population of young refugee orphans recently arrived in Portugal was evaluated. There is only one CACR in the country and represents a temporary shelter that constitutes a social response, which at the time of the study, was overcrowded (*n* = 16) for the theoretical capacity of 13 children. The purpose of this center is to provide specialized and transitional care for a maximum period of 6 months for children and young people under the age of 18 years [8]. Fifteen young people, eleven boys and five girls, aged between 16 and 18 years and from the following countries were considered valid for the present analysis: Guinea Conakry, Mali, Morocco, Mauritania, Nigeria, Republic of Congo, Senegal, and Sierra Leone.

### 2.3. Ethical Considerations

All research work was developed in accordance with the considerations contained in the Declaration of Helsinki [15]. The CACR and youth leader received detailed information about the project and signed an informed consent authorizing the youth to participate in the study. Data collection was only performed after confirming the informed consent and with the verbal consent of the young person obtained at the time of the anthropometric assessment.

### 2.4. Food Education Program

#### 2.4.1. Diagnosis

##### Initial Population Characteristics

Before the theoretical sessions began, an initial assessment session was carried out, and information was collected on the characteristics of the population through individual face-to-face interviews with each young person. With these interviews, information was collected on the nationality, food preferences in the country of origin, preferred foods in Portugal, date of arrival in Portugal, and religion of each young person.

##### Nutritional Knowledge

An initial nutritional knowledge questionnaire was developed by a nutritionist as part of the activity plan and applied to all participants. It consists of 38 closed-answer questions that address the themes of healthy food and habits. It was built using images and the possibility of traffic light responses. Before the questionnaire application, all images were explained in French and English. Some examples with questions and answers were also presented. This type of questionnaire was chosen because the participants had linguistic limitations in French and/or English, managing only to communicate in a very basic way. All participants completed the questionnaire and indicated that they had no doubts about completing it. The young orphan refugees had to indicate, using the image of a traffic light, what was their opinion about each image, since many of the young people could not read or write, and therefore a method perceptible by all was chosen. The traffic light had the colors red, yellow, and green, which respectively meant that it was something that you should not do or eat very often (red), that you can do or eat from time to time in a moderate way (yellow), and that you can do or eat more often but always moderately (green). Before being applied, the questionnaire was explained in French and English. The questionnaires were scored according to the percentage of correct answers: from 0% to 14%, 1 point was assigned; from 15% to 24%, 2 points; from 25% to 34%, 3 points; from 35% to 44%, 4 points; from 45% to 54%, 5 points; from 55% to 64%, 6 points; from 65% to 74%, 7 points; from 75% to 84%, 8 points; from 85% to 94%, 9 points; from 95% to 100%, 10 points.

##### Anthropometric Assessment

In the information collection session, an initial anthropometric assessment was also included. This assessment included weight, height, and waist circumference (WC). It should be noted that in addition to being carried out in the diagnostic phase, it was carried out after the educational intervention. The assessment of the weight and height of the young people was carried out according to the procedures recommended by the WHO/Europe, i.e., a weighing and two height measurements [16]. For the weight measurement, a SECA^®^ digital scale, model 840, with an accuracy of 100 g (Seca, Birmingham, UK) was used. The height was measured using a portable SECA^®^ brand stadiometer, model 214, with an accuracy of 1 mm (Seca, Birmingham, UK). The waist circumference was measured using a non-elastic measuring tape, as indicated in the WHO measurement protocol [16] at the midpoint between the last palpable rib and the top of the iliac crest. The Body Mass Index (BMI) was calculated according to the equation weight/(height^2^) (kg/m^2^). For the assessment of the nutritional status, the growth curves of the WHO were used for children aged 5 to 19 years [17], and in relation to the results of the Waist Circumference, its valuation was based on the distribution curve by age and sex presented in the Child and Youth Nutritional Status Assessment Guide [16].

#### 2.4.2. Intervention

The intervention phase consisted of nine nutritional education sessions with young people for 9 weeks (Table 1). The content of the sessions was based on the FAO manual “Eating well for good health” [18] which addresses the topics of nutrition, health, and healthy eating in a dynamic way, presenting materials for activities. Each session lasted an average of 2 h, and the strategies used during the session included reading in French and English what was written in the power point document that contained the theoretical content of each session, filling in small knowledge sheets proposed by the manual “Eating well for good health”, problem solving at the end of the sessions, and other dynamic activities. In addition to the sessions, the CACR monthly menu was also reviewed. Some nutritionally less balanced meals were identified, and suggestions were made for changes to meals that were nutritionally more appropriate for the age group of the participants and simultaneously according to their food preferences and tastes in Portugal and in their countries of origin (within what was financially possible for the Center). The main problems were: (1) an excessive supply of meals with fried foods and red meat and (2) a large amount of animal protein and little variety and supply of plant-based protein foods, such as legumes, cereals, and vegetables. Modifications were suggested with cooking recipes without fried foods, some of them vegetarian, and the daily supply of legumes and vegetables on the plate was encouraged. It was also recommended to introduce a vegetable soup on the daily menu and encourage the consumption of fruit at lunch and dinner, always putting this information on the weekly menu and remembering at mealtimes that there was fruit available for consumption after the main course.

#### 2.4.3. Evaluation

The evaluation of the nutritional intervention program was carried out by comparing the anthropometric indexes obtained in the initial and final anthropometric assessments and the scores of the nutritional knowledge questionnaires applied before and after the food education sessions. The final knowledge questionnaire presented 34 images of behaviors and foods, identical to the initial knowledge questionnaire, and the young people indicated the right option by selecting one of the colors of the traffic light, as previously described for the initial assessment. The final anthropometric assessment included weight, height, and WC as in the initial assessment and was performed using the same measurement techniques described above. The results of the initial and final nutritional knowledge evaluation of the young people were obtained and compared, as well as the anthropometric indexes. A possible association between the success of the intervention program and the attendance of each young person was also studied, verified by signing attendance sheets in each session.

## 3. Results

Table 2 presents the initial characteristics of the sample. Regarding the age of the young people, an average of 17 years was observed. The study was carried out on 15 young people, 11 of whom were male, and 4 were female. As for their nationalities, four were from Guinea Conakry, four from Congo, two from Sierra Leone, one from Nigeria, one from Morocco, one from Mali, one from Senegal, and one from Mauritania. Most had been in Portugal for less than a year but for more than 6 months (53.3%), only one had been in the center for more than a year, and the rest had lived in the center for less than 6 months (40.0%). Regarding the religious beliefs of the young people, 4 young people described themselves as being Christians, and the remaining 11 as Muslims.

Table 3 shows the food preferences of the young people in their countries of origin. The food that everyone preferred was rice. In addition to this, the food preferences of the young people were generally for the typical tubercules of each country and for the cereals available there. Some also mentioned vegetables, as is the case of the young people from Congo, Morocco, and Senegal. It was observed that beans were the legume chosen by the young people from Guinea Conakry, Sierra Leone, Nigeria, and Senegal. Various fruits were also referred to as favorite foods by the young people from Guinea Conakry, Congo, Nigeria, and Mali. Regarding meat, the young people from Sierra Leone and Senegal reported to prefer chicken.

Table 4 presents the foods preferred by young people in Portugal, divided by food groups. Regarding the group of cereals, derivatives, and tubercules, the favorite foods consumed in Portugal by young people are rice, potatoes, pasta, and couscous. The favorite vegetables are lettuce, carrots, and cucumbers. The fruit choices include mango, banana, apple, and tangerine. The preferred dairy products are milk and cheese and, in relation to the legumes group, the choice is beans.

Regarding the nutritional status criterion BMI-for-age, all the participants were between the 25th and the 50th percentile at the beginning of the Nutritional Intervention Program. According to Table 5, small changes were observed in the mean value of some anthropometric measurements at the end of the nine sessions of nutritional intervention. It was also found that there was, on average, an improvement in nutritional knowledge (+1.53 points) between the beginning and the end of the food education sessions.

Table 6 shows the differences in the scores between the initial and the final questionnaire and the attendance at the food education sessions. Of the 15 adolescents, 3 showed no difference in the scores of the nutritional knowledge questionnaires (0 points) and were present in an average of two sessions. On the other hand, four adolescents showed a difference of more than 1 point and attended an average of five and a half sessions. There were seven adolescents who scored 2 points from the initial questionnaire to the final questionnaire, and the average number of sessions attended was six. The only adolescent who attended all nine sessions also stood out for the maximum difference in the score with an improvement of three units. A greater attendance at the food education sessions seemed to favor the improvement of nutritional knowledge. The single youth (6.7%) who attended all nine sessions (100%) reported the biggest difference in the results with a 3-point increase. Of the seven young people (46.7%) who presented a difference of more than 2 points, six (40.0%) were present in more than five sessions (55.6%).

## 4. Discussion

To the best of our knowledge, this is the first study that assessed the possible beneficial contribution that a food education program may have on young refugees residing in Portugal. The evaluation and analysis of the results of this work are important to make improvements and choose more ways to approach young people, so that their nutritional literacy continues to increase, allowing them to gain independence and make autonomous and healthy choices. Several studies [2,19,20] have been performed on the contribution of education interventions with practical components, and there is evidence that shows differences in learning and knowledge acquisition, thus improving behaviors. In this way, holding food education sessions that combine a practical with a theoretical component may be an asset to the understanding and application of the knowledge acquired by young refugees.

At the end of the study, an improvement in nutritional knowledge was observed in the sample studied after the completion of nine sessions on food education, as well as a direct relationship between attendance at the sessions and improvement in nutritional knowledge. However, the sessions were not mandatory, which influenced the impact of the nutritional intervention, which could have been greater if the presence of some of the young people had been more frequent.

The language spoken during the sessions may have been one of the factors that contributed to the program not having even a greater impact. Most young people were learning to speak Portuguese, and there was not a common language for all. The sessions were in Portuguese so that the young people could get used to the language, but were also simultaneously translated into French and English, and all written documents handed out to the participants were either in French or in English, but sometimes were not fully understood by some of the participants.

The intervention had a duration of 12 weeks, and the food education sessions were carried out in 9 weeks, with an average duration of 2 h per week. The duration of the project may also have been a factor that affected the impact of the intervention, because longer and more frequent intervention periods could be more useful [21].

Regarding the nutritional status, no significant differences were observed in the young people. There are studies [22,23] that showed that even increased knowledge and, consequently, significant changes in the eating habits may not be accompanied by differences in people’s weight. The anthropometric index that showed the greatest difference was the abdominal circumference. This variation can be explained by the entry into Ramadan, a period in which young people abstain from food during the day and only eat at night. Since the measurements were taken at two in the afternoon, at this time the young people of Muslim religion would not have eaten since the day before. This value can also be justified by the dietary change to which the young people were subjected. Bearing in mind that, in Portugal, access to protein foods is easier than in their countries of origin, this greater consumption of proteins may have helped to reduce the abdominal fat, as shown in several studies [24,25] when replacing large amounts of foods rich in carbohydrates with some more protein-rich foods.

During the nutritional intervention, suggestions were made to modify the CACR monthly menu, with the aim of providing the young people with more nutritionally balanced meals, considering their food preferences. The suggested changes to the menus have not yet been put into practice; therefore, the evaluation of their success and acceptance was not included in this study.

Although most of the young people’s food was dependent on the monthly food availability of the center, some young people also had access to other nutritionally unbalanced foods. The Western diet to which they were exposed had a negative impact on their food habits, since in their countries of origin they did not usually eat highly processed foods, rich in salt, simple sugars, and saturated trans fats. As for other displaced populations previously studied [26], it was found that the young people at the Shelter for Refugee Children sometimes ate a less healthy diet than they did in their country of origin, although the access to food is simpler in Portugal.

The present study has some limitations: (1) communication was challenging due to the language difficulties of the participants and the behaviors of distrust and insecurity at the beginning of the program; (2) the impossibility of the CACR institution to immediately implement all the changes suggested in the monthly menu due to financial limitations; (3) the short length of stay at the temporary shelter, which made it difficult to implement a longer nutritional intervention program with more nutrition education sessions. In the future, it will be important to establish a permanent nutritional intervention program so that this vulnerable group of the population displaced to Portugal can be educated on healthy and safe food.

We hope to be able to replicate the education program at later stages and involve other young refugees who will be hosted by the Centre, so that in the future we will have a higher number of cases on which to evaluate its effectiveness on nutritional knowledge. Some of the cases that did not participate in at least half of the educational sessions or that voluntarily decided not to participate in any of the sessions, will be integrated into the control group to which only the questionnaire and anthropometric evaluations will be carried out. 

## 5. Conclusions

The increase in nutrition knowledge is a crucial factor for changing eating habits and preventing obesity. The present work showed that food education programs for young refugees residing in Portugal can improve their nutritional knowledge and favor their integration into the Portuguese society and culture. This food education program contributed to a better understanding by young orphan refugees newly arrived in Portugal of the foods available in Portugal and of the Portuguese gastronomy.

In the future, the more assiduous presence of young people should be an objective to be achieved; thus, there is a need to find strategies to entice their participation through even more dynamic, interesting, and age-appropriate activities. More visual techniques should also be used, considering the lack of a common language for all young people. The duration and frequency of the sessions should also be greater, so that there would be a constant monitoring and a greater possibility of carrying out the sessions in a more personal way, thus verifying the difficulties of each young person and the strategies to be applied to increase the understanding and acquisition of nutritional knowledge. Also involving the entire community of the center in the sessions will be an asset in the future for the impact of the implemented program, especially the employees directly responsible for feeding the young people. This nutritional and food education program functioned as a pilot study, and the results showed the need to continue it. Improving the methodology of the food education program and increasing its impact on young CACR refugees are future goals.

## Figures and Tables

**Table 1:** Plan for food and nutrition education sessions.

Sessions	Theoretical Component	Practical Component
1	What does it mean to be healthy and well nourished?	Filling in a form.
2	Fundamental conditions for a good state of Nutrition and Malnutrition.	Filling in a form.
3	What do we get from Food? Vitamins and Minerals.	Filling in a form and preparing natural juices.
4	What do we get from Food? Carbohydrates.	Filling in a form.
5	What do we get from Food? Proteins.	Preparation of “Cloud Bread”.
6	What do we get from Food? Lipids.	Preparation of homemade mayonnaise.
7	Food Safety and Labeling.	Label Analysis.
8	Food Waste and Food Guides Around the World.	Presentation of each young person’s country food guides.
9	Healthy Choices–Home Economics.	Go to the supermarket to prepare a shopping list for a week with EUR 20.

**Table 2:** Initial characteristics of the sample.

Features	Adolescents (*n* = 15)
Age years	17.0 ± 0.6
Sex	
Female	4 (26.7%)
Male	11 (73.3%)
Nationality	
Guinea Conakry	4 (26.7%)
Congo	4 (26.7%)
Sierra Leone	2 (13.3%)
Nigeria	1 (6.70%)
Morocco	1 (6.70%)
Mali	1 (6.70%)
Senegal	1 (6.70%)
Mauritania	1 (6.70%)
Length of stay in Portugal	
>12 months	1 (6.70%)
>6 and <12 months	8 (53.3%)
<6 months	6 (40.0%)
Religion	
Muslim	11 (73.3%)
Christian	4 (26.7%)

Results are expressed as number (percentage) or mean ± standard deviation.

**Table 3:** Food preferences in the countries of origin.

Country of Origin	Foods
Guinea Conakry	Corn, rice, cassava, potato, beans, banana bread, mango, avocado, orange, and coconut.
Congo	Rice, cassava, banana and vegetables.
Sierra Leone	Rice, couscous, spaghetti, cassava, beans, and chicken.
Nigeria	Rice, sweet potato, cassava, beans, and melon.
Morocco	Rice, couscous, and vegetables.
Mali	Rice, potato, and banana bread.
Senegal	Rice, vegetables, beans, chicken, and peanuts.
Mauritania	Rice and corn.

**Table 4:** Food preferences in Portugal.

Food Groups	Foods
Derived Cereals and Tubers	Rice, Potato, Pasta couscous
Vegetables	Lettuce, Carrot Cucumber
Fruit	Mango, Banana, Litter, Tangerine
Dairy Products and Derivatives	Cheese, Milk
Legumes	Beans

**Table 5:** Comparison of the anthropometric measurements and scores obtained in the assessment of nutritional knowledge before and after the intervention sessions.

Anthropometric Measures	Initial Results	Final Results	Difference
Weight (kg)	64.72 ± 9.95	64.69 ± 9.65	−0.01 ± 1.09
BMI (kg/m^2^)	21.20 ± 2.91	21.26 ± 2.91	+0.03 ± 0.35
WC (cm)	74.86 ± 5.27	74.00 ± 5.31	−0.86 ± 1.08
Nutritional Knowledge Score	4.06 ± 1.09	5.60 ± 1.12	+1.53 ± 0.83

Results are expressed as mean ± standard deviation. BMI, Body Mass Index; WC, Waist Circumference.

**Table 6:** Difference in the scores of the nutritional knowledge questionnaires and in attendance at the food education sessions.

Adolescents (*n* = 15)	Initial Questionnaire Score	Final Questionnaire Score	Difference	Attendance at the Sessions	Attending at Least 5 Sessions	Improved Questionnaire Score
1	6	6	+0	3 (33.3%)	0	0
2	4	5	+1	5 (55.6%)	1	1
3	3	4	+1	4 (44.4%)	0	1
4	5	6	+1	5 (55.6%)	1	1
5	3	5	+2	8 (88.9%)	1	1
6	4	6	+2	8 (88.9%)	1	1
7	2	4	+2	3 (33.3%)	0	1
8	4	4	+0	2 (22.2%)	0	0
9	4	6	+2	8 (88.9%)	1	1
10	4	7	+3	9 (100%)	1	1
11	3	5	+2	5 (55.6%)	1	1
12	6	8	+2	5 (55.6%)	1	1
13	4	6	+2	7 (77.8%)	1	1
14	4	5	+1	8 (88.9%)	1	1
15	6	6	+0	1 (11.1%)	0	0

Results are expressed as a number or number (percentage).

## Data Availability

Not applicable.
